# Author Correction: Regulation of the one carbon folate cycle as a shared metabolic signature of longevity

**DOI:** 10.1038/s41467-021-24840-z

**Published:** 2021-08-12

**Authors:** Andrea Annibal, Rebecca George Tharyan, Maribel Fides Schonewolff, Hannah Tam, Christian Latza, Markus Max Karl Auler, Sebastian Grönke, Linda Partridge, Adam Antebi

**Affiliations:** 1 Max Planck Institute for Biology of Ageing, Cologne, Germany; 2 Cologne Excellence Cluster on Cellular Stress Responses in Aging-Associated Diseases (CECAD), University of Cologne, Cologne, Germany

**Keywords:** Metabolomics, Molecular biology

Correction to: *Nature Communications* 10.1038/s41467-021-23856-9, published online 09 June 2021

The original version of this Article omitted from the author list the 7th and 8th authors Sebastian Grönke and Linda Partridge, respectively, who are from the Max Planck Institute for Biology of Ageing, Cologne, Germany. Consequently, the following was added to the Author contributions: “S.G. and L.P. generated the transgenic mouse line and provided the tissue sample for the targeted metabolomic analysis.”

Further, this article contained an error in the final paragraph of the “Results” section, in the sentence “Targeted metabolomic analysis of folic acid intermediates using brain and liver tissues from male *Irs1*−/− knockout mice revealed a profile similar to worm daf-2 mutants (Fig. 5c, e and Supplementary Table 6).” In fact, female mice were used.

In the Methods section and the Reporting Summary the animal experiment permit number was wrongly stated as VSG 84-02.04.2013.A158. The correct permit number is VSG 84-02.04.2014.A215. And the mouse strain used was C3B6F1, not C57BL/6, as incorrectly stated. The sentences “C57BL/6 *Irs1*−/− KO mice were originally obtained from the lab of Prof. Dominic Withers’ lab (Imperial College, London). These mice were then backcrossed into the C3H/HeOuJ backkground by Marker-Assisted Accelerated Backcrossing (MAX-BAX®) conducted by Charles River. In order to generate homozygous C3B6F1 hybrid *Irs1*−/− KO mice, C3H/HeOuJ *Irs1*−/+ females were mated with males of the C57BL/6 *Irs1*−/+ KO strain. To generate C3B6F1 wild type control mice, C3H/HeOuJ females were mated with C57BL/6NCrl males (strain codes 626 and 027, respectively, Charles River Laboratories). All mice were bred on-site at the mouse facility of the Max Planck Institute for Biology of Ageing, Cologne. The *Irs1*−/− KO mice were homozygous and only females were used for the experiments.” were added to the Methods section and the Reporting Summary.

These errors have now been corrected in the PDF and HTML versions of the article.

