# Supplementary material for: Regulation of the one carbon folate cycle as a shared metabolic signature of longevity
Source: Nat Commun. 2021 Jun 9;12:3486. doi: 10.1038/s41467-021-23856-9 (PMC8190293; doi:10.1038/s41467-021-23856-9)
Supplement: Supplementary file 1 — Supplementary Information [file 41467_2021_23856_MOESM1_ESM.pdf]

Supplementary Information

Regulation of the one carbon folate cycle as a shared metabolic signature of longevity

Authors: Andrea Annibal[1], 1Rebecca George Tharyan[1] ,Maribel Schoenewolff[1], Hannah Tam[1], Christian Latza[1], Markus Max Karl Auler[1] Adam Antebi[1,2,3]

1Max Planck Institute for Biology of Ageing, Joseph Stelzmann Str. 9b, 50931, Cologne, Germany

2Cologne Excellence Cluster on Cellular Stress Responses in Aging-Associated Diseases (CECAD), University of Cologne, 50931, Cologne, Germany

3Corresponding author

Supplementary Fig. 1

a

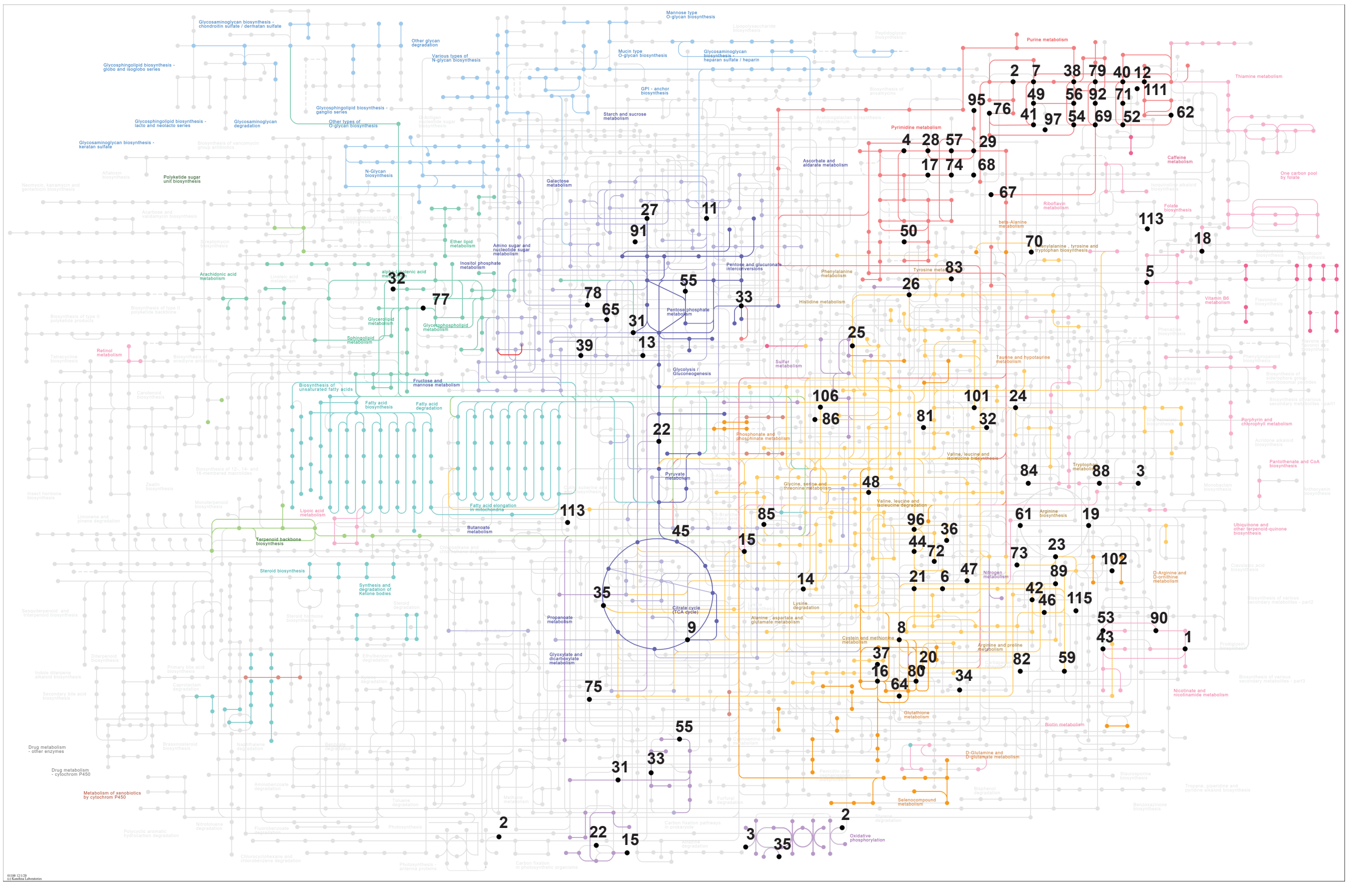

b

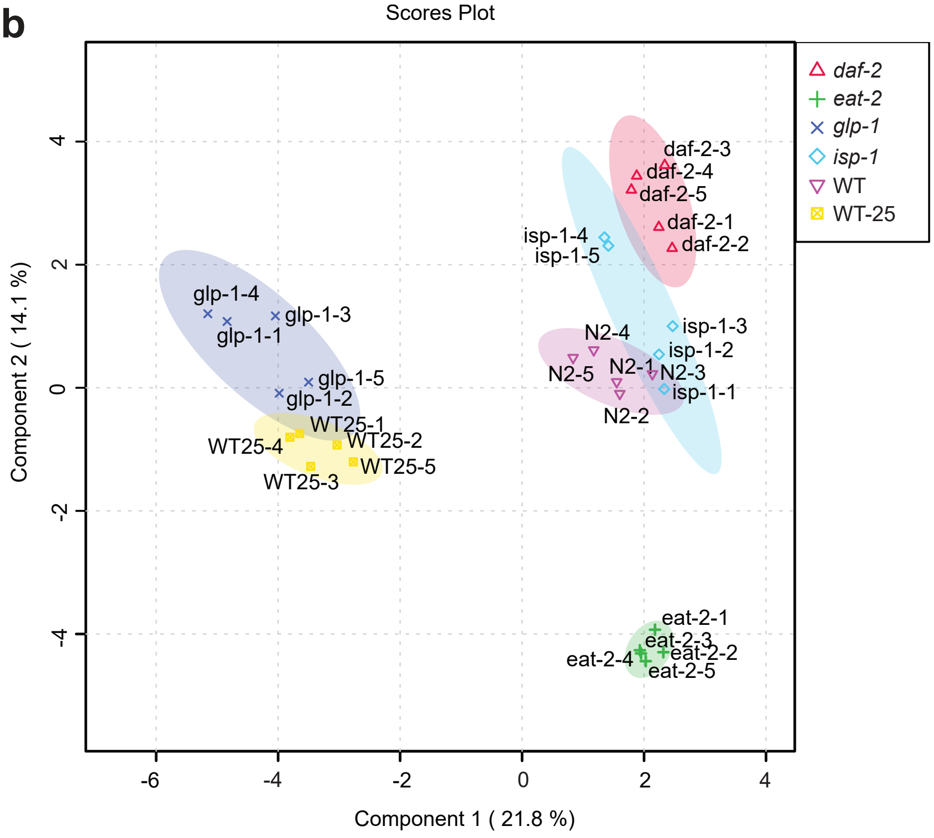

Supplementary Fig. 1

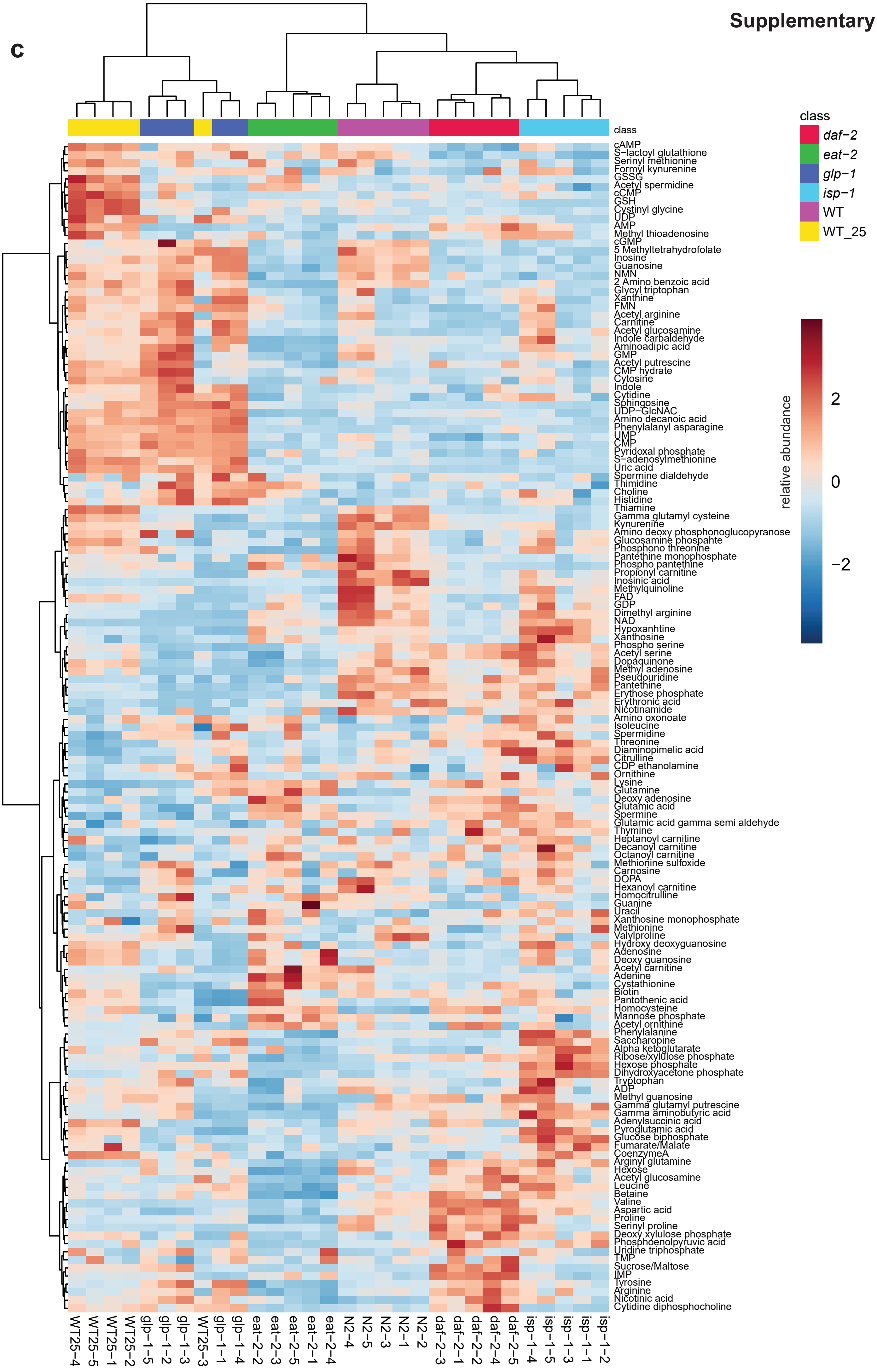

Supplementary Fig. 1

d

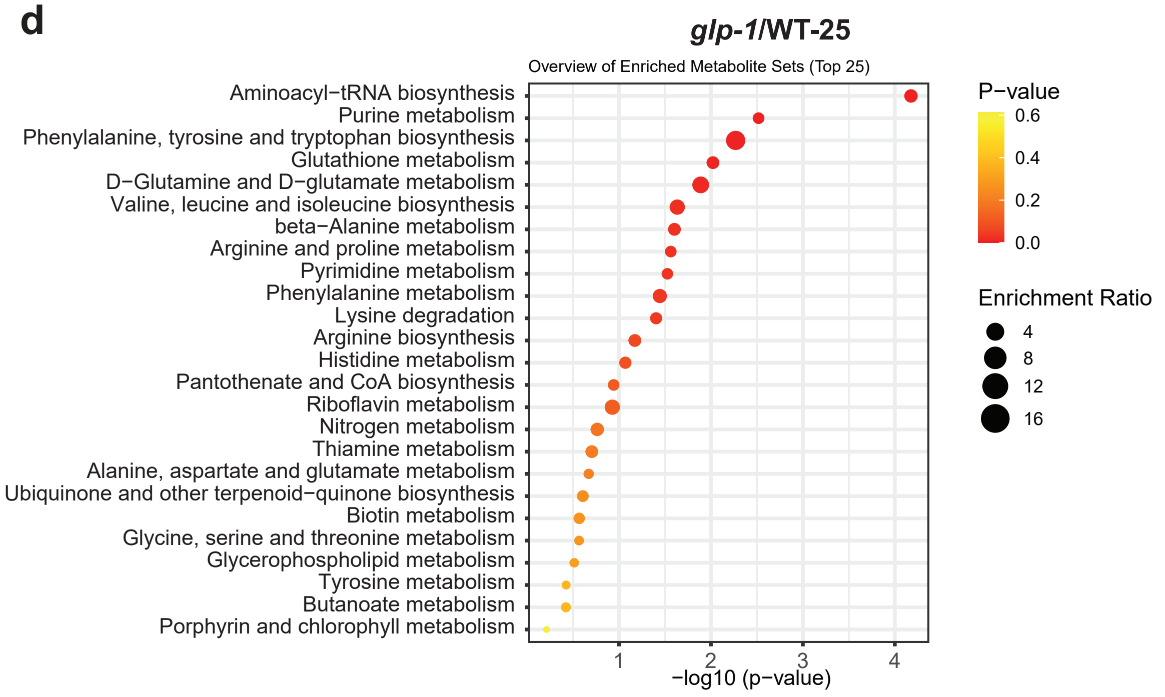

e

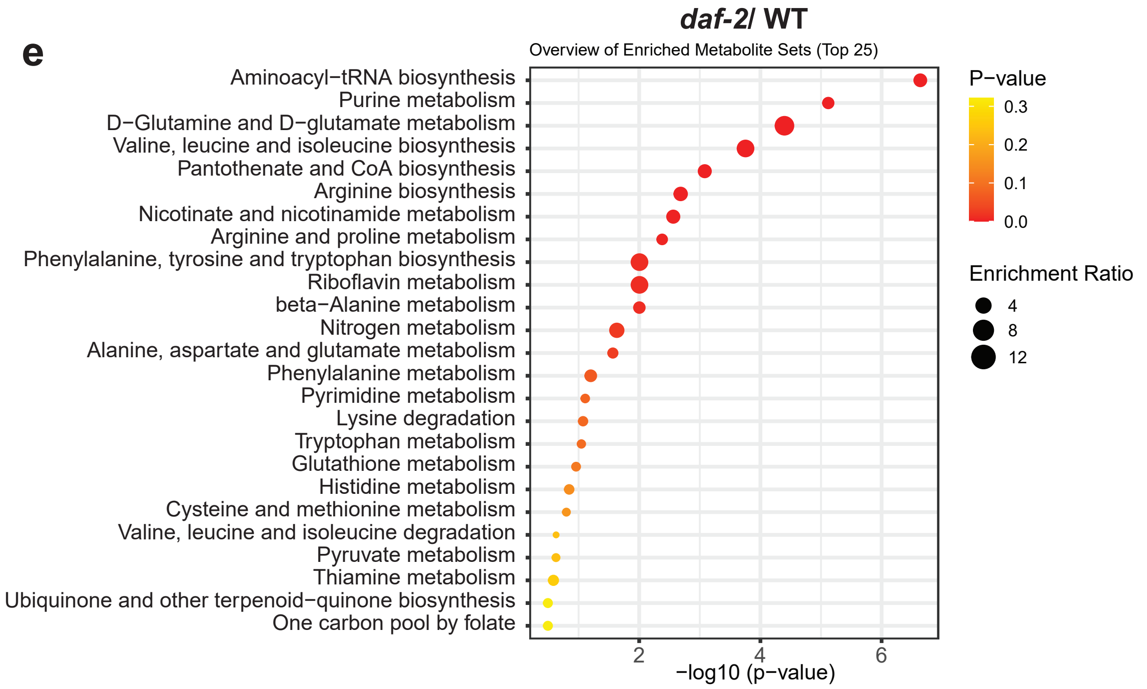

Supplementary Fig. 1

f

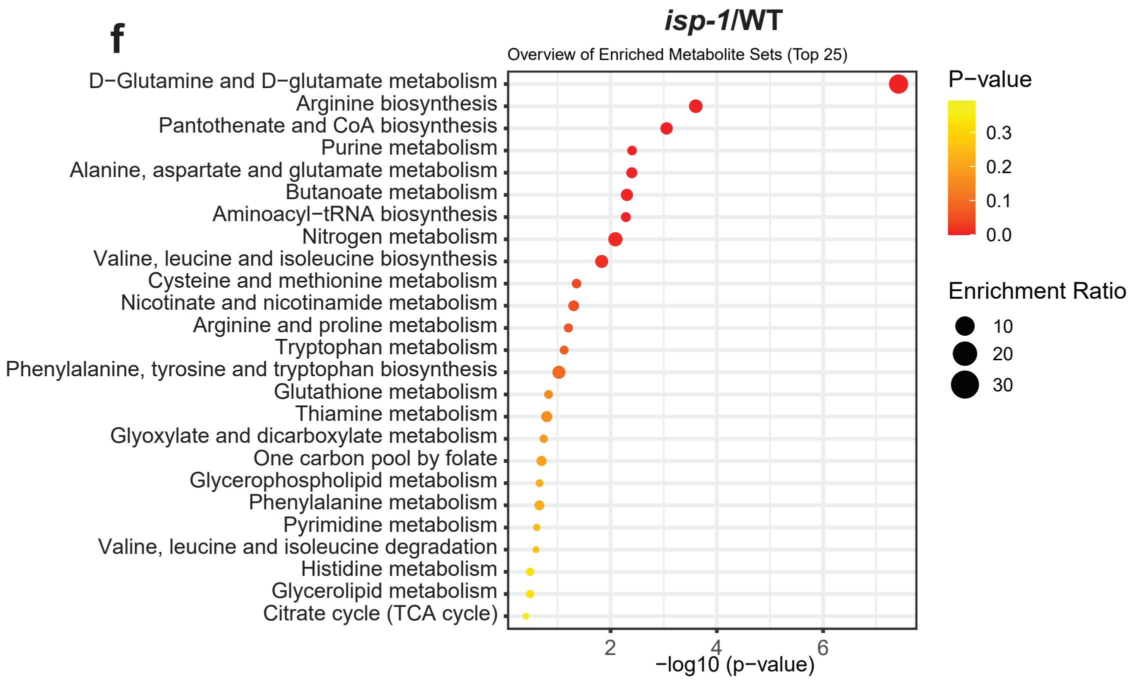

g

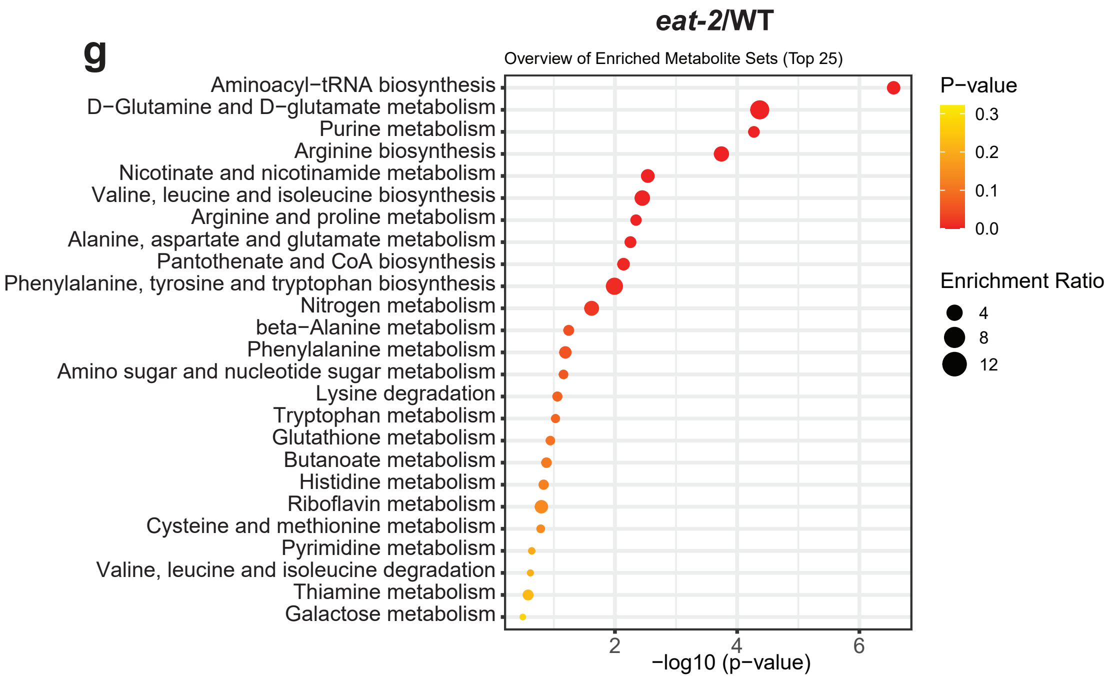

Supplementary Fig. 2

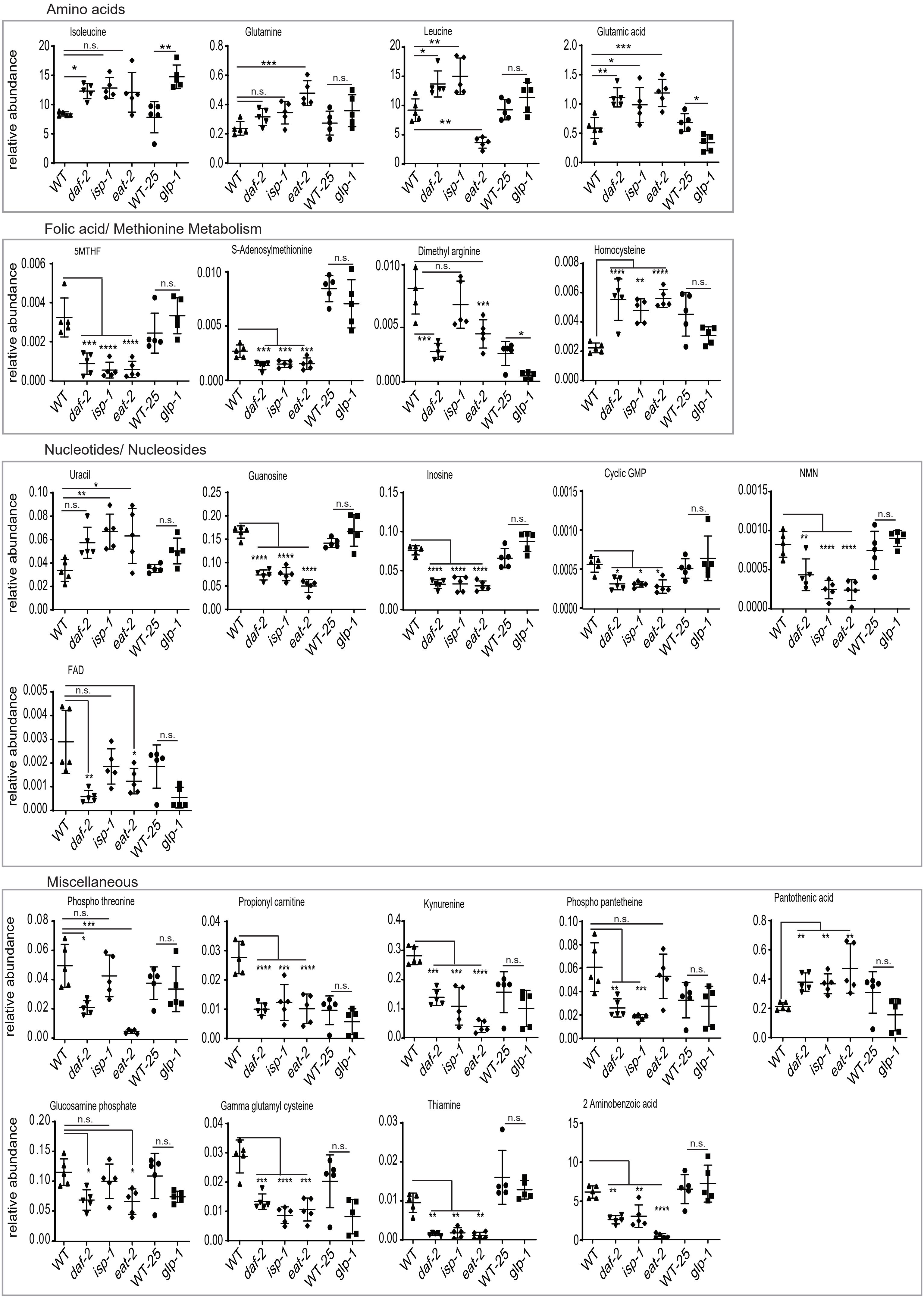

Supplementary Fig 3

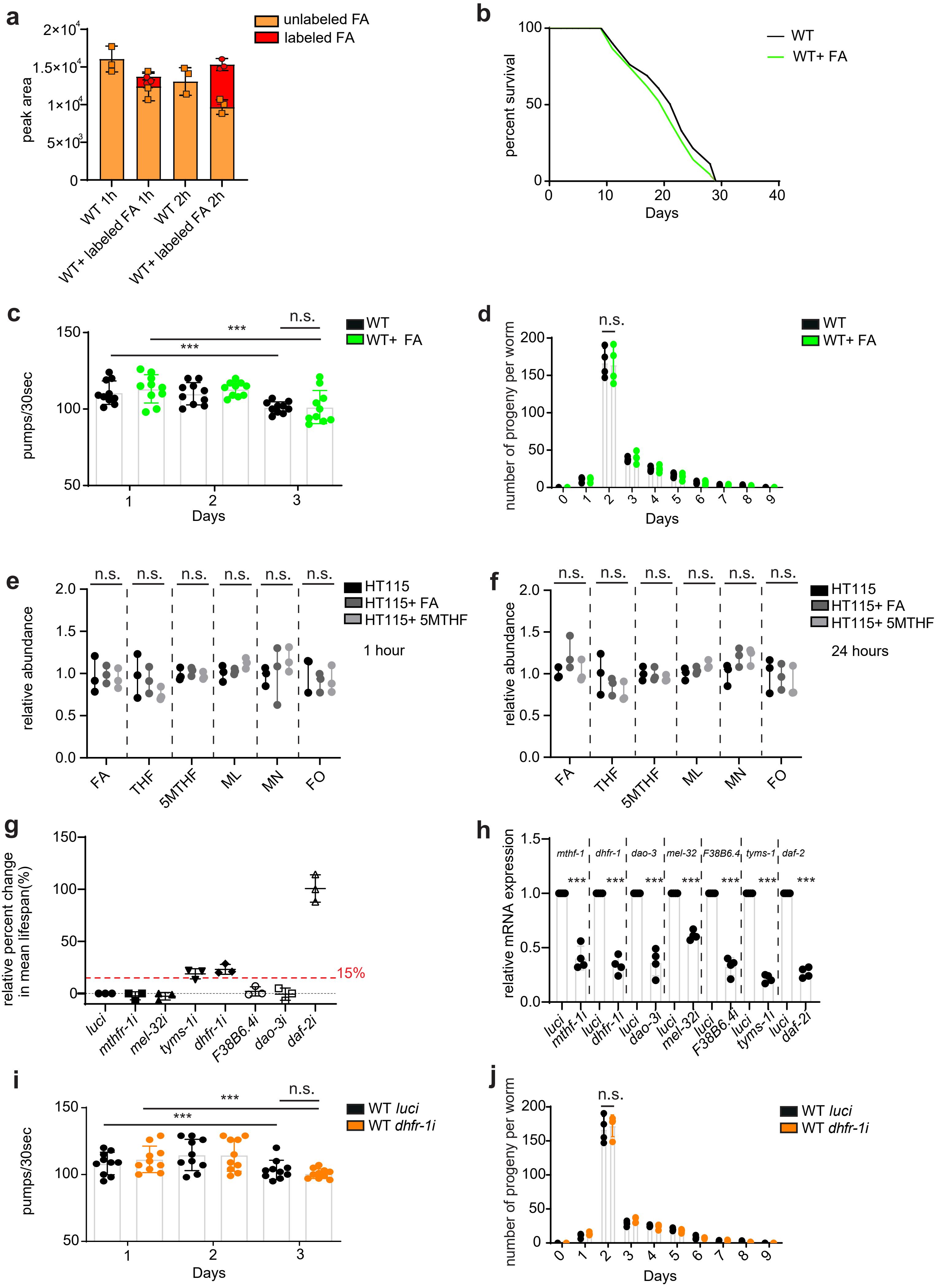

Supplementary Fig. 4

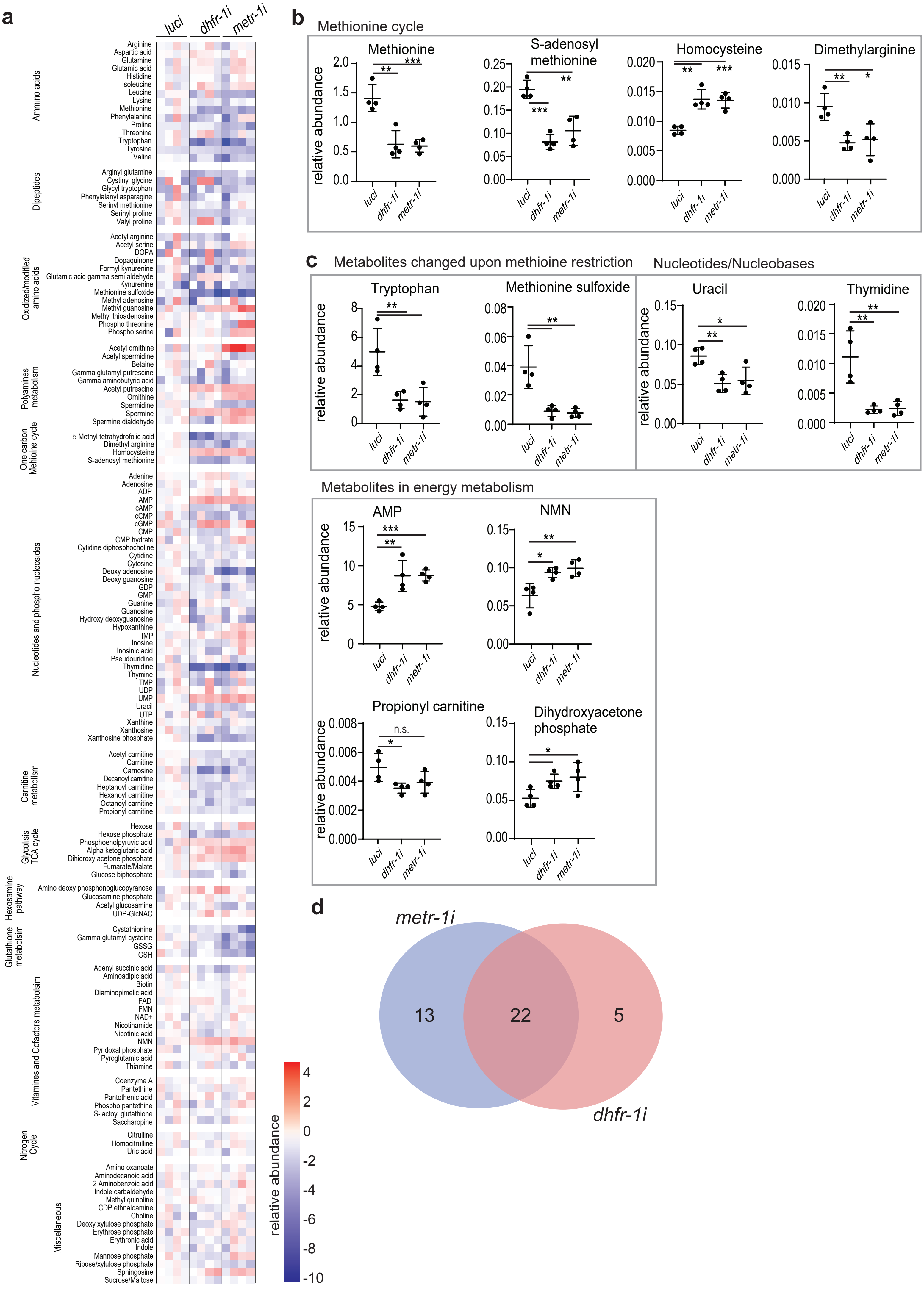

Supplementary Fig. 4

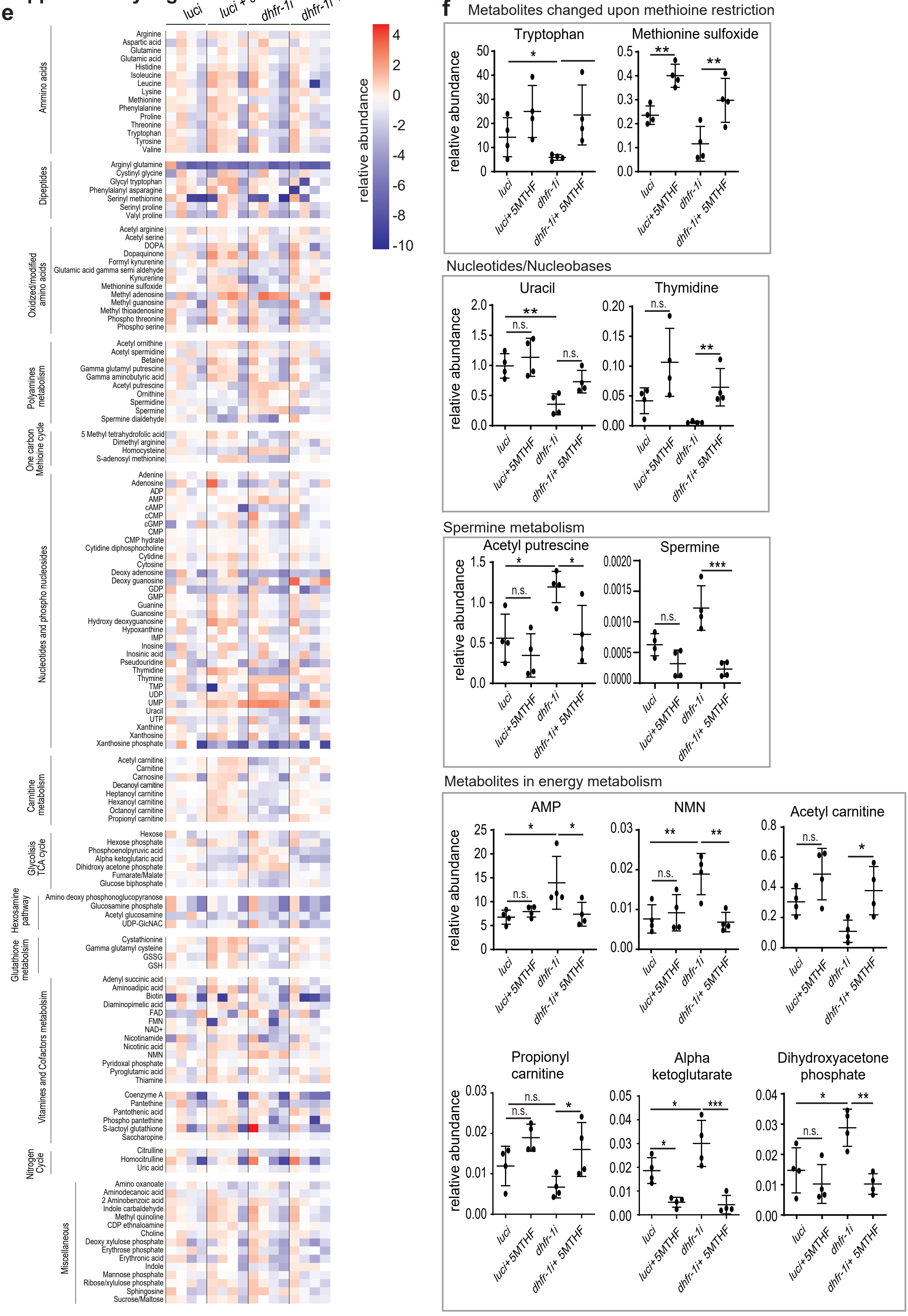

Supplementary Fig 5

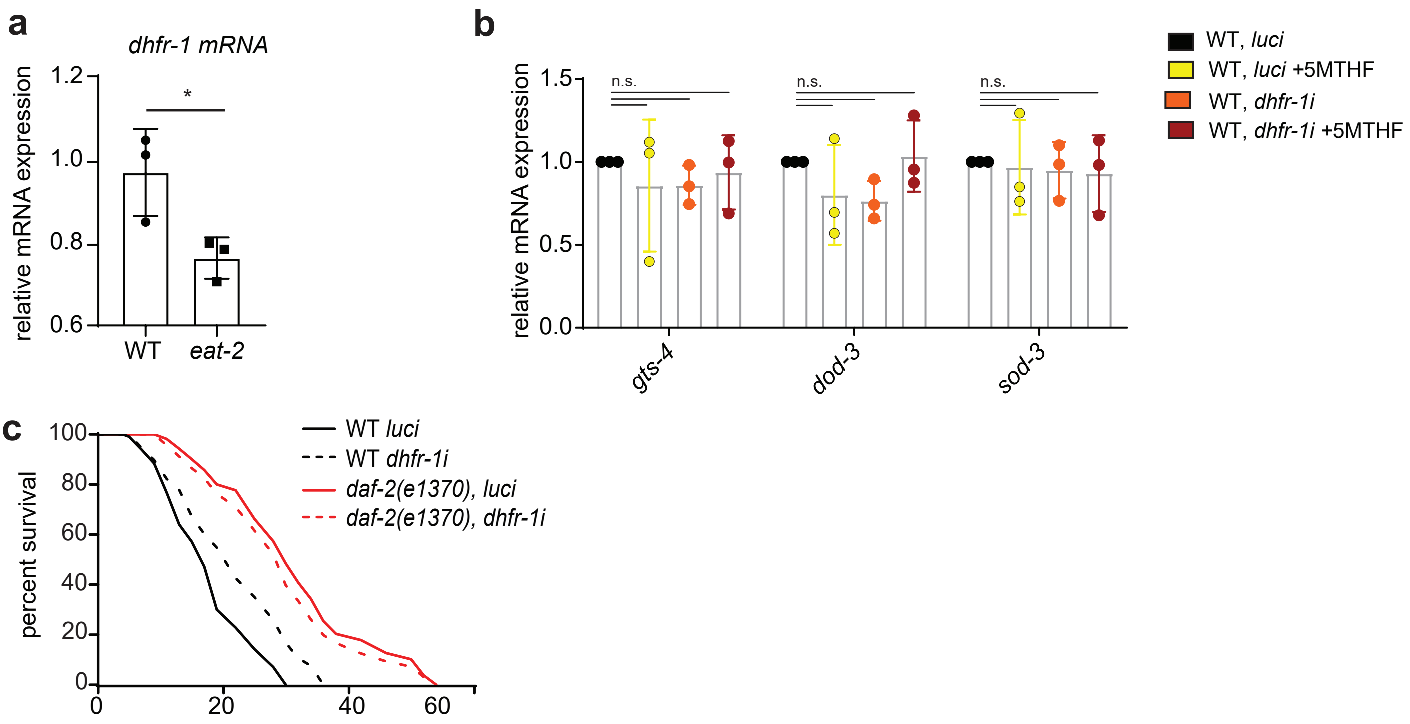

Supplementary Figure 1: Untargeted metabolomics and pathway analysis of longevity mutants (linked to Fig.1).

a, Mapping of identified metabolites onto KEGG metabolic pathways. Each number refers to a compound listed in Supplementary Data 2. **b,** PLS-DA scores of the untargeted metabolomic features of all five conditions obtained by MetaboAnalyst (https://www.metaboanalyst.ca). Component 1, X axis, Component 2, Y axis. In the score plot, the ellipse corresponds to 95% confidence region. **c,** Hierarchical cluster heat maps obtained from MetaboAnalyst using the following parameters: Distance measure, Euclidian, Cluster algorithm Ward. Normalized data were converted into log2 scale. **d-g**, Enrichment analysis of significant metabolites (adj p<.05) obtained by uploading differentially regulated metabolites to MetaboAnalyst (https://www.metaboanalyst.ca) in the various mutants compared to WT. KEGG pathways containing 84 metabolite sets (KEGG, Oct. 2019) was selected. Detailed parameters are shown in Supplementary Table 3.

Supplementary Figure 2: Differentially regulated metabolites in the longevity mutants (linked to Fig. 1).

Significant metabolites (adj p<.05) for *daf-2, isp-1* and *eat-2* genotypes are selected from the untargeted metabolomics analysis (Fig.1a, Supplementary Data 1) and plotted. *glp-1* and WT-25°C are shown for reference. Significance was assessed using one-way anova Dunnett's multiple comparisons test * p<0.5, ** p<0.01, ***p<0.001. Data are presented as mean ± S.D.

Supplementary Figure 3: Effect of folic acid supplementation and folic acid cycle gene knockdown on worm physiology and lifespan (linked to Fig. 2,3,4).

a, Incorporation of $^{13}C^{15}N$ labeled folic acid in WT worms measured by targeted metabolomics. At 1 and 2 hours, the concentration of labeled FA increases without affecting the overall folic acid concentration pool.

b, Supplementation of 10nM folic acid does not affect WT lifespan (from L4 stage) **c,** pharyngeal pumping over 30 second period (day 1 adult) and **d,** brood size of self-fertilizing hermaphrodites during adulthood. **e,f,** Targeted metabolomic analysis of folic acid cycle intermediates in HT115 bacteria upon folic acid and 5MTHF supplementation for 1 hour (**e**) or 24 hr (**f**) show no significant difference in steady state levels of these intermediates.

g, Mini-RNAi screen for life span of genes involved in the folic acid cycle (from L4 stage). *dhfr-1i* and *tyms-1i* showed an increase in mean lifespan >15%. **h,** RNAi knockdown efficiency of folate cycle genes (day 1 adult). **i,** Pharyngeal pumping measurement over 30 seconds in *luci* control and *dhfr-1i* treated worms (day 1). **j,** Brood size of *luci* control and *dhfr-1i* treated worms*.* Self-fertilizing brood size was measured during adulthood.

a, N=3 independent biological replicates, each dot represent a single replicate. **b,** n=150 worms per repeat per conditions, N=3 independent biological replicates. **c,i,** n=15 worms, N=3 independent biological replicates. Each dot represent a single worm. **d,j,** n=10 worms, N=4 independent biological replicates. Each dot represent the average of eggs from 10 worms. **e-h,** N=3 independent biological replicates.

b,g Statistics were analyzed by two-sided Mantel–Cox log-rank test (Supplementary Table 7 for statistics). **c,e,f,i,** Significance was assessed using one-way anova Dunnett's

multiple comparisons test * p<0.5, ** p<0.01, ***p<0.001. **d,h,j** Significance was assessed using two-sided t-test. Bar shows mean ± S.D, * p<0.5, ** p<0.01, ***p<0.001.

Supplementary Figure 4. Untargeted metabolomic analysis comparing *dhfr-1i* and *metr-1i*, and *dhfr-1i* with or without 5MTHF supplementation (linked to Fig 2,3).
a, Heat map depicting untargeted metabolomic analysis of *luci, dhfr-1i* and *metr-1i*, (day 1 of adult) manually grouped in different categories. Metabolite concentrations are listed in Supplementary Data 2.
b, Significantly changed metabolites (p<.05) from the methionine cycle are selected from the untargeted metabolomics analysis and plotted.
c, Significantly changed metabolites (p<.05) associated with methionine restriction.
d, Venn diagram showing the unique and shared significant metabolites (p<.05) between *metr-1i* and *dhfr-1i*.
The unique metabolites for *dhfr-1i* (p<.05) are serinyl proline, hexanoyl carnitine, 5MTHF, octanoyl carnitine, propionyl carnitine. The unique metabolites of *metr-1i* (p<.05) are spermidine, ribose/xylulose phosphate, alpha ketoglutarate, arginyl glutamine, valine, ornithine, cystathionine, GSH, gamma glutamyl cysteine, IMP, GSSG, acetyl ornithine, cytidine diphosphocholine. The twenty-two metabolites in common (p<.05) are thymidine, AMP, uracil, hexose phosphate, spermine, heptanoyl carnitine, cAMP, spermine dialdehyde, acetyl carnitine, deoxy adenosine, homocysteine, dimethyl arginine, UMP, S-adenosylmethionine, tryptophan, methionine sulfoxide, xanthosine phosphate, acetyl putrescine, NMN, dihydroxyacetone phosphate, methionine, formyl kynurenine.
e, Heat map of targeted metabolomic analysis of *luci* control and *dhfr-1i* with and without 10nM 5MTHF supplementation (day 1 of adult), manually grouped in different categories. Metabolite concentrations are listed in Supplementary Data 2. **f,** Significant metabolites (p<.05) associated with methionine restriction are selected from the untargeted metabolomics analysis and plotted.
a,e, N=4 independent biological replicates. **a,e** Normalized metabolites concentrations are converted to log2 for heat map generation **a,e,** Statistics were determined using the one-sided Fisher test and Benjamini-Hochberg correction (p<.05) for multiple comparison **b,c,f** Significance was assessed using one-way anova Dunnett's multiple comparisons test. * p<0.5, ** p<0.01, ***p<0.001. Data are presented as mean ± S.D.

Supplementary Figure 5. Regulation of methionine restriction and folate cycle associated genes.
a, *dhfr-1* mRNA expression is downregulated in *eat-2* relative to wild type based on RNA-seq data from Heestand 2013 (day 1 adult). **b,** *daf-16* dependent target gene expression in wild type worms with *luci* or *dhfr-1i* in the presence or absence of 5MTHF (day 1 adult). No significant (n.s.) difference was found between the conditions. **c,** *dhfr-1i* (from L4 stage) does not affect *daf-2(e1370)* lifespan.
a,b, N=3 independent biological replicates. **c,** n=150 worms per repeat per conditions, N=2 independent biological replicates. **a,b,** Significance was assessed using two-sided t-test. Bar shows mean ± S.D, * p<0.5, ** p<0.01, ***p<0.001. **c,** Statistics were analyzed by two-sided Mantel–Cox log-rank test (Supplementary Table 7 for statistics).

Supplemental Table Summary

Supplemental Table 1: List of compounds mapped to KEGG metabolic pathways (Linked to Extended Data Fig 1a).

Supplemental Table 2: Comparison of metabolomics analysis with previous published works.

Supplemental Table 3: Enrichment analysis of metabolites in longevity mutants (linked to Extended Data Fig 1d,e,f,g).

Supplemental Table 4: Features submitted to PIUMet (linked to Fig. 1c).

Supplemental Table 5: PIUMet output and input (linked to Fig. 1c).

Supplemental Table 6: Targeted metabolomic analysis of folates and methionine.

Supplemental Table 7: Lifespan Analyses**.**

Supplemental Table 8: List of gene activation upon methionine restriction validated by qRT-PCR according to Tang *et al* (linked to Figure 3d, Figure 5b).

Supplemental Table 9: Strains used in this study.

Supplemental Table 10: qRT-PCR primers

Supplemental Table 1: List of compounds mapped to KEGG metabolic pathways (Linked to Extended Data Fig 1a).

Number	KEGG identifier	Compound name
1	C00003	NAD+
2	C00008	ADP
3	C00010	CoA
4	C00015	UDP
5	C00018	Pyridoxal phosphate
6	C00019	S-adenosyl methionine
7	C00020	AMP
8	C00025	Glutamate
9	C00026	Malate
11	C00031	Glucose
12	C00035	GDP
13	C00043	UDP-GlcNAC
14	C00047	Lysine
15	C00049	Aspartic acid
16	C00051	GSH
17	C00055	CMP
18	C00061	FMN
19	C00062	Arginine
20	C00064	Glutamine
21	C00073	Methionine
22	C00074	Phosphoenolpyruvic acid
23	C00077	Ornithine
24	C00078	Tryptophan
25	C00079	Phenylalanine
26	C00082	Tyrosine
27	C00103	Glucose phosphate
28	C00105	UMP
29	C00106	Uracil
31	C00111	Glycerol phosphate
32	C00114	Choline
33	C00117	Ribose/xylulose phosphate
34	C00120	Biotin
35	C00122	Fumarate
36	C00123	Leucine
37	C00127	GSSG
38	C00130	IMP

39	C00140	Acetyl glucosamine
40	C00144	GMP
41	C00147	Adenine
42	C00148	Proline
43	C00153	NMN
44	C00155	Homocysteine
46	C00170	Methyl thio adenosine
47	C00183	Valine
48	C00188	Threonine
49	C00212	Adenosine
50	C00214	Thymidine
52	C00242	Guanine
53	C00253	Nicotinic acid
54	C00262	Hypoxanthine
55	C00279	Erythronic acid
56	C00294	Inosine
57	C00299	Uridine
59	C00315	Spermidine
61	C00327	Citrulline
62	C00330	Deoxy guanosine
64	C00334	Amino oxonoate
65	C00352	Glucosamine phosphate
67	C00366	Uric acid
68	C00380	Cytosine
69	C00385	Xanthine
70	C00386	Carnosine
71	C00387	Guanosine
72	C00407	Isoleucine
73	C00437	Acetyl ornithine
74	C00475	Cytidine
75	C00489	glutaric acid
76	C00559	Deoxy adenosine
77	C00570	CDP ethanolamine
78	C00636	Mannose phosphate
79	C00655	Xanthosine 5 P
80	C00669	Gamma glutamyl cysteine
81	C00719	Betaine
82	C00750	Spermine
83	C00822	Dopaquinone
84	C00864	Pantothenic acid
85	C00956	Aminoadipic acid

86	C00979	Acetyl serine
88	C01134	Pantethine phosphate
89	C01165	Glutamate semialdehyde
90	C01185	Nicotinamide
91	C01231	Glucose bisphosphate
92	C01762	Xanthosine
95	C02067	Pseudouridine
96	C02291	Cytidine diphosphocholine
97	C02353	cAMP
101	C02700	Formyl kynurenine
102	C02714	Acetyl putrescine
103	C02989	Methionine oxide
106	C03508	Amino butanoate
111	C06194	cGMP
113	C11437	Deoxy xylulose phosphate
115	C15699	Gamma glutamyl putrescine

Supplemental Table 2: Comparison of metabolomics analysis with previous published works.

1 Wan QL, Shi X, Liu J, Ding AJ, Pu YZ, Li Z, Wu GS, Luo HR. Metabolomic signature associated with reproduction-regulated aging in Caenorhabditis elegans. Aging (Albany NY). 2017 Feb 6;9(2):447-474. doi: 10.18632/aging.101170. PMID: 28177875; PMCID: PMC5361674.

2 Fuchs S, Bundy JG, Davies SK, Viney JM, Swire JS, Leroi AM. A metabolic signature of long life in Caenorhabditis elegans. BMC Biol. 2010 Feb 10;8:14. doi: 10.1186/1741-7007-8-14. PMID: 20146810; PMCID: PMC2829508.

3 Pontoizeau C, Mouchiroud L, Molin L, Mergoud-Dit-Lamarche A, Dallière N, Toulhoat P, Elena-Herrmann B, Solari F. Metabolomics analysis uncovers that dietary restriction buffers metabolic changes associated with aging in Caenorhabditis elegans. J Proteome Res. 2014 Jun 6;13(6):2910-9. doi: 10.1021/pr5000686. Epub 2014 May 22. PMID: 24819046; PMCID: PMC4059273.

4 Butler JA, Mishur RJ, Bhaskaran S, Rea SL. A metabolic signature for long life in the Caenorhabditis elegans Mit mutants. Aging Cell. 2013 Feb;12(1):130-8. doi: 10.1111/acel.12029. PMID: 23173729; PMCID: PMC3552119.

5 Gao AW, Smith RL, van Weeghel M, Kamble R, Janssens GE, Houtkooper RH. Identification of key pathways and metabolic fingerprints of longevity in C. elegans. Exp Gerontol. 2018 Nov;113:128-140. doi: 10.1016/j.exger.2018.10.003. Epub 2018 Oct 6. PMID: 30300667; PMCID: PMC6224709.

6 Hastings J, Mains A, Virk B, Rodriguez N, Murdoch S, Pearce J, Bergmann S, Le Novère N, Casanueva O. Multi-Omics and Genome-Scale Modeling Reveal a Metabolic Shift During C. elegans Aging. Front Mol Biosci. 2019 Feb 6;6:2. doi: 10.3389/fmolb.2019.00002. PMID: 30788345; PMCID: PMC6372924.

Red upregulated, blue down regulated, only significant metabolites are compared.

We indicate the term AGREE when a metabolite from our work changes in the same direction from published works. We indicate the term DISAGREE when a metabolite from our work did not change or changed in the opposite direction from published works.

	This study				1	2	3	4	5	6
compounds name	daf-2/WT	eat-2/WT	isp-1/WT	glp-1/WT 25	glp-1/WT	daf-2/WT	eat-2/WT	isp-1/WT	daf-2/WT eat-2/WT	WT aging
2 Aminobenzoic acid										
5 Methyl tetrahydrofolate										
Acetyl arginine										
Acetyl carnitine										DISAGREE
Acetyl glucosamine										
Acetyl glucosamine										
Acetyl ornithine										
Acetyl serine										
Acetyl spermidine										
Acetyl putrescine										
Adenine					AGREE				AGREE	
Adenosine					AGREE					
Adenylsuccinic acid										
ADP										
Alpha ketoglutarate										
Amino adipic acid										DISAGREE
Amino decanoic acid										
Amino deoxyphosphono glucopyranose										
Amino oxonoate										
AMP					AGREE				AGREE	
Arginine					AGREE	DISAGREE	AGREE	AGREE		AGREE
Arginyl glutamine										
Aspartic acid						DISAGREE			AGREE	
Betaine						DISAGREE				AGREE
Biotin					DISAGREE					
cAMP										
Carnitine					AGREE					DISAGREE

Carnosine										
cCMP										
CDP ethanolamine					AGREE					
cGMP										
Choline					AGREE					DISAGREE
Citrulline										
CMP										
CMP hydrate										
CoenzymeA										
Cystathionine										
Cystinyl glycine										
Cytidine diphosphocholine							AGREE			AGREE
Cytidine					AGREE					AGREE
Cytosine					DISAGREE					
Decanoyl carnitine										
Deoxy adenosine										
Deoxy guanosine										
Deoxy xylulose phosphate										
Diaminopimelic acid										
Dihydroxyacetone phosphate										
Dimethyl arginine										
DOPA										
Dopaquinone										
Erythronic acid										
Erythrose phosphate										
FAD										
FMN										
Formyl kynurenine										

Fumarate/Malate						DISAGREE		AGREE		
Gamma amino butyric acid										
Gamma glutamyl cysteine										
Gamma glutamyl putrescine										
GDP										
Glucosamine phosphate										
Glucose bisphosphate										
Glutamic acid					AGREE	DISAGREE	DISAGREE	AGREE		AGREE
Glutamic acid gamma semialdehyde										
Glutamine					DISAGREE		AGREE	AGREE	AGREE	
Glycyl tryptophan										
GMP										DISAGREE
GSH					AGREE					
GSSG										
Guanine										
Guanosine										DISAGREE
Heptanoyl carnitine										
Hexanoyl carnitine										
Hexose										
Hexose phosphate					DISAGREE				DISAGREE	
Histidine					AGREE					
Homocitrulline										
Homocysteine										
Hydroxy deoxy guanosine										
Hypoxanthine								DISAGREE		
IMP					DISAGREE					
Indole										

Indole carbaldehyde										
Inosine										
Inosinic acid										
Isoleucine					AGREE	AGREE	DISAGREE	AGREE	AGREE	
Kynurenine										
Leucine						AGREE	AGREE		AGREE	AGREE
Lysine						AGREE	AGREE	AGREE		AGREE
Mannose phosphate										
Methionine								AGREE	DISAGREE	DISAGREE
Methionine sulfoxide										
Methyl adenosine										
Methyl guanosine										
Methyl quinoline										
Methyl thioadenosine										
NAD+									DISAGREE	
Nicotinamide										
Nicotinic acid						DISAGREE				
NMN										
Octanoyl carnitine										
Ornithine										
Pantethine										
Pantothenic acid										
Phenylalanine										AGREE
Phenylalanyl asparagine										
Phospho serine										
Phosphoenolpyruvic acid										
Phospho threonine										
Phospho pantethine										
Proline						DISAGREE	AGREE	AGREE	AGREE	

Propionyl carnitine										
Pseudouridine										
Pyridoxal phosphate										
Pyroglutamic acid										
Ribose/xylulose phosphate										
Saccharopine										
S-adenosylmethionine					DISAGREE					
Serinyl methionine										
Serinyl proline										
S-lactoylglutathione										
Spermidine										
Spermine										
Spermine dialdehyde										
Sphingosine										
Sucrose/Maltose										
Thiamine										
Threonine								AGREE	DISAGREE	DISAGREE
Thymidine					DISAGREE					
Thymine										
TMP										
Tryptophan						AGREE				DISAGREE
Tyrosine					AGREE	AGREE	AGREE	AGREE		
UDP										
UDP-GlcNAC										
UMP									DISAGREE	
Uracil								DISAGREE		DISAGREE
Uric acid										
Uridine triphosphate										
Valine						AGREE	AGREE	DISAGREE		

Valylproline								AGREE		
Xanthine								AGREE	DISAGREE	
Xanthosine										
Xanthosine-5-phosphate										

Supplemental Table 3: Enrichment analysis of metabolites in longevity mutants (linked to Extended Data Fig 1d,e,f,g).

glp-1 vs WT-25	total	expected	hits	Raw p	Holm p	FDR
Aminoacyl-tRNA biosynthesis	48	1.48	8	6.67E-05	0.0056	0.0056
Purine metabolism	65	2.01	7	0.00303	0.251	0.127
Phenylalanine, tyrosine and tryptophan biosynthesis	4	0.124	2	0.00538	0.442	0.151
Glutathione metabolism	28	0.865	4	0.00949	0.769	0.199
D-Glutamine and D-glutamate metabolism	6	0.185	2	0.0129	1	0.217
Valine, leucine and isoleucine biosynthesis	8	0.247	2	0.0232	1	0.278
beta-Alanine metabolism	21	0.648	3	0.0249	1	0.278
Arginine and proline metabolism	38	1.17	4	0.0273	1	0.278
Pyrimidine metabolism	39	1.2	4	0.0297	1	0.278
Phenylalanine metabolism	10	0.309	2	0.0359	1	0.301
Lysine degradation	25	0.772	3	0.0394	1	0.301
Arginine biosynthesis	14	0.432	2	0.0671	1	0.47
Histidine metabolism	16	0.494	2	0.0851	1	0.55
Pantothenate and CoA biosynthesis	19	0.587	2	0.114	1	0.661
Riboflavin metabolism	4	0.124	1	0.118	1	0.661
Nitrogen metabolism	6	0.185	1	0.172	1	0.902
Thiamine metabolism	7	0.216	1	0.197	1	0.976
Alanine, aspartate and glutamate metabolism	28	0.865	2	0.213	1	0.995
Ubiquinone and other terpenoid-quinone biosynthesis	9	0.278	1	0.247	1	1
Biotin metabolism	10	0.309	1	0.27	1	1
Glycine, serine and threonine metabolism	33	1.02	2	0.271	1	1
Glycerophospholipid metabolism	36	1.11	2	0.306	1	1
Tyrosine metabolism	42	1.3	2	0.375	1	1
Butanoate metabolism	15	0.463	1	0.377	1	1
Porphyrin and chlorophyll metabolism	30	0.926	1	0.613	1	1
Glyoxylate and dicarboxylate metabolism	32	0.988	1	0.637	1	1

Cysteine and methionine metabolism	33	1.02	1	0.649	1	1
Amino sugar and nucleotide sugar metabolism	37	1.14	1	0.691	1	1
Fatty acid degradation	39	1.2	1	0.71	1	1
Valine, leucine and isoleucine degradation	40	1.24	1	0.72	1	1

daf-2 vs WT	total	expected	hits	Raw p	Holm p	FDR
Aminoacyl-tRNA biosynthesis	48	2.02	12	2.30E-07	1.93E-05	1.93E-05
Purine metabolism	65	2.73	12	7.59E-06	0.00063	0.000319
D-Glutamine and D-glutamate metabolism	6	0.252	4	4.01E-05	0.00329	0.00112
Valine, leucine and isoleucine biosynthesis	8	0.336	4	0.000176	0.0142	0.00369
Pantothenate and CoA biosynthesis	19	0.799	5	0.000828	0.0662	0.0139
Arginine biosynthesis	14	0.589	4	0.00207	0.164	0.029
Nicotinate and nicotinamide metabolism	15	0.631	4	0.00274	0.214	0.0329
Arginine and proline metabolism	38	1.6	6	0.00418	0.322	0.0439
Phenylalanine, tyrosine and tryptophan biosynthesis	4	0.168	2	0.00989	0.752	0.0758
Riboflavin metabolism	4	0.168	2	0.00989	0.752	0.0758
beta-Alanine metabolism	21	0.883	4	0.00993	0.752	0.0758
Nitrogen metabolism	6	0.252	2	0.0234	1	0.164
Alanine, aspartate and glutamate metabolism	28	1.18	4	0.0272	1	0.176
Phenylalanine metabolism	10	0.42	2	0.063	1	0.378
Pyrimidine metabolism	39	1.64	4	0.0777	1	0.435
Lysine degradation	25	1.05	3	0.0844	1	0.443
Tryptophan metabolism	41	1.72	4	0.0899	1	0.444
Glutathione metabolism	28	1.18	3	0.11	1	0.514
Histidine metabolism	16	0.673	2	0.143	1	0.633
Cysteine and methionine metabolism	33	1.39	3	0.159	1	0.666

Valine, leucine and isoleucine degradation	40	1.68	3	0.235	1	0.9
Pyruvate metabolism	22	0.925	2	0.236	1	0.9
Thiamine metabolism	7	0.294	1	0.26	1	0.95
Ubiquinone and other terpenoid-quinone biosynthesis	9	0.378	1	0.321	1	1
One carbon pool by folate	9	0.378	1	0.321	1	1
Vitamin B6 metabolism	9	0.378	1	0.321	1	1
Biotin metabolism	10	0.42	1	0.35	1	1
Glyoxylate and dicarboxylate metabolism	32	1.35	2	0.393	1	1
Amino sugar and nucleotide sugar metabolism	37	1.56	2	0.467	1	1
Butanoate metabolism	15	0.631	1	0.477	1	1
Tyrosine metabolism	42	1.77	2	0.535	1	1
Citrate cycle (TCA cycle)	20	0.841	1	0.579	1	1
Glycolysis / Gluconeogenesis	26	1.09	1	0.676	1	1
Porphyrin and chlorophyll metabolism	30	1.26	1	0.728	1	1
Glycine, serine and threonine metabolism	33	1.39	1	0.761	1	1
Glycerophospholipid metabolism	36	1.51	1	0.791	1	1

isp-1 vs WT	total	expected	hits	Raw p	Holm p	FDR
D-Glutamine and D-glutamate metabolism	6	0.146	5	3.80E-08	3.19E-06	3.19E-06
Arginine biosynthesis	14	0.34	4	0.000249	0.0207	0.0105
Pantothenate and CoA biosynthesis	19	0.462	4	0.000884	0.0725	0.0247
Purine metabolism	65	1.58	6	0.00394	0.319	0.0618
Alanine, aspartate and glutamate metabolism	28	0.681	4	0.00399	0.319	0.0618
Butanoate metabolism	15	0.365	3	0.00492	0.389	0.0618
Aminoacyl-tRNA biosynthesis	48	1.17	5	0.00515	0.402	0.0618
Nitrogen metabolism	6	0.146	2	0.00811	0.625	0.0852

Valine, leucine and isoleucine biosynthesis	8	0.194	2	0.0147	1	0.137
Cysteine and methionine metabolism	33	0.802	3	0.0437	1	0.367
Nicotinate and nicotinamide metabolism	15	0.365	2	0.0495	1	0.378
Arginine and proline metabolism	38	0.924	3	0.0622	1	0.436
Tryptophan metabolism	41	0.997	3	0.0748	1	0.484
Phenylalanine, tyrosine and tryptophan biosynthesis	4	0.0972	1	0.0938	1	0.563
Glutathione metabolism	28	0.681	2	0.147	1	0.821
Thiamine metabolism	7	0.17	1	0.159	1	0.832
Glyoxylate and dicarboxylate metabolism	32	0.778	2	0.181	1	0.896
One carbon pool by folate	9	0.219	1	0.199	1	0.919
Glycerophospholipid metabolism	36	0.875	2	0.217	1	0.919
Phenylalanine metabolism	10	0.243	1	0.219	1	0.919
Pyrimidine metabolism	39	0.948	2	0.245	1	0.969
Valine, leucine and isoleucine degradation	40	0.972	2	0.254	1	0.969
Histidine metabolism	16	0.389	1	0.327	1	1
Glycerolipid metabolism	16	0.389	1	0.327	1	1
Citrate cycle (TCA cycle)	20	0.486	1	0.391	1	1
Fructose and mannose metabolism	20	0.486	1	0.391	1	1
beta-Alanine metabolism	21	0.511	1	0.406	1	1
Pyruvate metabolism	22	0.535	1	0.42	1	1
Lysine degradation	25	0.608	1	0.462	1	1
Glycolysis / Gluconeogenesis	26	0.632	1	0.475	1	1
Inositol phosphate metabolism	30	0.729	1	0.526	1	1
Porphyrin and chlorophyll metabolism	30	0.729	1	0.526	1	1
Fatty acid degradation	39	0.948	1	0.622	1	1

eat-2 vs WT	total	expected	hits	Raw p	Holm p	FDR
Aminoacyl-tRNA biosynthesis	48	2.05	12	2.75E-07	2.31E-05	2.31E-05
D-Glutamine and D-glutamate metabolism	6	0.256	4	4.27E-05	0.00355	0.00149

Purine metabolism	65	2.78	11	5.32E-05	0.00436	0.00149
Arginine biosynthesis	14	0.598	5	0.000181	0.0147	0.0038
Nicotinate and nicotinamide metabolism	15	0.641	4	0.0029	0.232	0.0487
Valine, leucine and isoleucine biosynthesis	8	0.342	3	0.00357	0.282	0.05
Arginine and proline metabolism	38	1.62	6	0.00452	0.353	0.0542
Alanine, aspartate and glutamate metabolism	28	1.2	5	0.00558	0.429	0.0586
Pantothenate and CoA biosynthesis	19	0.811	4	0.00724	0.55	0.0676
Phenylalanine, tyrosine and tryptophan biosynthesis	4	0.171	2	0.0102	0.765	0.0856
Nitrogen metabolism	6	0.256	2	0.0241	1	0.184
beta-Alanine metabolism	21	0.897	3	0.0571	1	0.399
Phenylalanine metabolism	10	0.427	2	0.0648	1	0.417
Amino sugar and nucleotide sugar metabolism	37	1.58	4	0.0695	1	0.417
Lysine degradation	25	1.07	3	0.0875	1	0.49
Tryptophan metabolism	41	1.75	4	0.094	1	0.493
Glutathione metabolism	28	1.2	3	0.114	1	0.563
Butanoate metabolism	15	0.641	2	0.132	1	0.616
Histidine metabolism	16	0.683	2	0.147	1	0.649
Riboflavin metabolism	4	0.171	1	0.16	1	0.655
Cysteine and methionine metabolism	33	1.41	3	0.164	1	0.655
Pyrimidine metabolism	39	1.67	3	0.23	1	0.88
Valine, leucine and isoleucine degradation	40	1.71	3	0.242	1	0.884
Thiamine metabolism	7	0.299	1	0.264	1	0.923
Galactose metabolism	27	1.15	2	0.322	1	1
Ubiquinone and other terpenoid-quinone biosynthesis	9	0.384	1	0.326	1	1
One carbon pool by folate	9	0.384	1	0.326	1	1
Biotin metabolism	10	0.427	1	0.355	1	1
Glyoxylate and dicarboxylate metabolism	32	1.37	2	0.401	1	1
Glycine, serine and threonine metabolism	33	1.41	2	0.416	1	1
Glycosylphosphatidylinositol (GPI)-anchor biosynthesis	14	0.598	1	0.459	1	1

Tyrosine metabolism	42	1.79	2	0.544	1	1
Citrate cycle (TCA cycle)	20	0.854	1	0.585	1	1
Fructose and mannose metabolism	20	0.854	1	0.585	1	1
Pentose phosphate pathway	22	0.94	1	0.62	1	1
Pyruvate metabolism	22	0.94	1	0.62	1	1
Glycolysis / Gluconeogenesis	26	1.11	1	0.682	1	1
Porphyrin and chlorophyll metabolism	30	1.28	1	0.733	1	1
Glycerophospholipid metabolism	36	1.54	1	0.796	1	1

Supplemental Table 4: Features submitted to PIUMet (linked to Fig. 1c).

Molecular weight	Ion mode	Retention time
304.2038	positive	13.279
515.3015	positive	16.277
547.3632	positive	21.297
545.3481	positive	18.407
571.3638	positive	12.865
473.2481	positive	9.193
505.3173	positive	18.214
503.3008	positive	17.255
501.2798	positive	9.157
499.2689	positive	20.962
529.3117	positive	12.401
302.1882	positive	13.22
451.2699	positive	21.071
479.3007	positive	16.53
477.2855	positive	7.941
691.5157	positive	20.782
733.5593	positive	20.893
783.5728	positive	21.425
777.5297	positive	19.306
430.3083	positive	12.689
761.5826	positive	20.933
689.498	positive	17.538
687.4834	positive	20.952
737.4953	positive	20.323

705.5304	positive	18.118
767.5449	positive	21.292
797.5915	positive	20.94
795.5716	positive	21.057
713.4955	positive	20.185
787.512	positive	20.737
120.0299	positive	2.14
727.55	positive	21.141
288.1725	positive	2.609
300.2089	positive	11.492
584.4441	positive	12.035
552.4748	positive	10.553
752.6327	positive	21.721
519.3207	positive	17.227
517.3163	positive	16.946
132.0774	negative	12.903
132.0775	negative	13.467
475.2696	negative	16.709
529.3168	negative	13.26
481.3168	negative	21.589
477.2855	negative	17.28
221.1048	negative	13.533
219.1103	negative	9.062
731.5445	negative	20.746
745.5598	negative	19.505
717.5306	negative	21.273
731.5793	negative	20.77
316.2191	negative	12.071
230.0192	negative	13.141
336.074	negative	8.142
536.0445	negative	2.428
147.0532	negative	3.493
515.3104	negative	13.023
543.3418	negative	13.435
541.3285	negative	17.104
567.3442	negative	17.645

Supplemental Table 5: PIUMet output and input (linked to Fig. 1c).

PIUmet output

Summary of the results, for the following parameters:
w=10.0 , beta=2.0, mu=0.0005, Da=0.01, R=1
The number of input terminals #peaks=25 , #Metabolites=0, #genes/proteins=0
155 peaks are matched to 162 metabolites in HMDB and Recon
5 peaks are matched to 52 metabolites in the PPMI network
The resulting network info:
-Number of nodes=21
-Number of edges=20
-Number of m/z peaks in the results=3
-Number of terminal metabolites in the results=0
-Number of terminal genes/proteins in the results=0

Result node frequency		
Node name	Robustness score	Identifier
BTD	1.0	Protein
Acetoacetic acid	1.0	Metabolite_Endogenous HMDB00060
Thymidine	1.0	Metabolite_Endogenous HMDB00273
DLD	1.0	Protein
SLC16A1	1.0	Protein
L-Valine	1.0	Metabolite_Food HMDB00883
m/z=102.02699	1.0	m/z Peak
Tetrahydrofolic acid	1.0	Metabolite_Endogenous HMDB01846
L-Lysine	1.0	Metabolite_Food HMDB00182
BCAT1	1.0	Protein
SLC6A14	1.0	Protein
TYMS	1.0	Protein
FPGS	1.0	Protein
TK2	1.0	Protein
m/z=242.0980966	1.0	m/z Peak
Biotin	1.0	Metabolite_Food HMDB00030
m/z=130.05816	1.0	m/z Peak
3-Methyl-2-oxovaleric acid	1.0	Metabolite_Endogenous HMDB00491
5-Thymidylic acid	1.0	Metabolite_Endogenous HMDB01227
Dihydrofolic acid	1.0	Metabolite_Endogenous HMDB01056

Result edge frequency	robustness score	confidence score from PPI
L-Lysine (pp) BTD	1.0	0.99167591126
L-Lysine (pp) SLC6A14	1.0	1

Tetrahydrofolic acid (pp) FPGS	1.0	1
Tetrahydrofolic acid (pp) DLD	1.0	0.960227059495
Thymidine (pp) TK2	1.0	0.956973829155
Thymidine (pp) m/z=242.0980966	1.0	0.969173076845
Dihydrofolic acid (pp) TYMS	1.0	1
Dihydrofolic acid (pp) FPGS	1.0	0.992540291593
DLD (pp) 3-Methyl-2-oxovaleric acid	1.0	1
TYMS (pp) 5-Thymidylic acid	1.0	1
3-Methyl-2-oxovaleric acid (pp) m/z=130.05816	1.0	0.960673820791
SLC6A14 (pp) L-Valine	1.0	1
BCAT1 (pp) 3-Methyl-2-oxovaleric acid	1.0	1
BCAT1 (pp) L-Valine	1.0	1
m/z=130.05816 (pp) 3-Methyl-2-oxovaleric acid	1.0	0.955639980233
Biotin (pp) SLC16A1	1.0	1
Biotin (pp) BTD	1.0	1
SLC16A1 (pp) Acetoacetic acid	1.0	1
5-Thymidylic acid (pp) TK2	1.0	0.98263263268
m/z=102.02699 (pp) Acetoacetic acid	1.0	1

Supplemental Table 6: Targeted metabolomic analysis of folates and methionine.

In each table, significance values are indicated below peak measurements.

**Figure 1d**					
	**Average peak area normalized to control (WT)**				
**Compounds**	**WT**	***daf-2(e1370)***	***isp-1(qm150)***	***eat-2(ad465)***	***glp-1(e2141ts)***
FA	1.0000	3.9657	1.8359	2.2170	0.9646
THF	1.0000	5.2470	1.8451	1.9509	1.4456
5MTHF	1.0000	0.4155	0.5234	0.3232	1.1651
MN	1.0000	1.4457	3.5562	3.2902	1.2650
ML	1.0000	0.1228	0.7649	0.5316	1.2557
FO	1.0000	0.6266	1.3589	0.4206	0.8029
Significance was assessed using one-way anova Dunnett's multiple comparisons test. * p<0.5, ** p<0.01, ***p<0.001					
**Compounds**		***daf-2(e1370) vs WT***	***isp-1(qm150) vs WT***	***eat-2(ad465) vs WT***	***glp-1(e2141ts) vs WT***
FA		0.0017	0.003	0.0029	0.9060
THF		0.0002	0.019	0.0254	0.0211
5MTHF		0.0002	0.0291	0.0002	0.2447
ML		0.0303	0.0033	<0.0001	0.2571
MN		<0.0001	0.0987	0.0032	0.3388

FO		0.0012	0.1126	0.0274	0.3756	
**Figure 2c**						
	**Average peak area normalized to control (WT)**					
**Compounds**	**Luciferase**	**Luciferase +10nM FA**	**Luciferase +10nM 5MTHF**	***dhfr-1i***	***dhfr-1i* +10nM 5MTHF**	***dhfr-1i+ FA***
FA	1.0000	0.9621	1.0330	2.0318	1.8614	2.0724
THF	1.0000	1.1318	2.1130	<LOD	1.1314	<LOD
5MTHF	1.0000	0.7106	1.5118	0.4364	0.9588	0.5186
MN	1.0000	0.8732	1.4748	0.3445	0.9179	0.6020
ML	1.0000	1.0180	1.0430	0.5353	0.9549	0.4592
FO	1.0000	0.7715	0.8714	0.4964	0.7587	0.4572
Significance was assessed using one-way anova Dunnett's multiple comparisons test. * p<0.5, ** p<0.01, ***p<0.001						
**Compounds**		**Luciferase vs +10nM FA**	**Luciferase vs +10nM 5MTHF**	**Luciferase vs *dhfr-1i***	***dhfr-1i* vs *dhfr-1i* +10nM 5MTHF**	**Luciferase vs *dhfr-1i+* FA**
FA		0.0001	0.0001	0.0001	0.6577	<0.0001
THF		0.7449	0.0001	N.D	N.D	N.D.
5MTHF		0.0405	0.0004	0.0001	0.0003	0.0035
MN		0.3897	0.0001	0.0001	0.0001	0.0059
ML		0.9997	0.9925	0.0015	0.0038	<0.0001
FO		0.1707	0.6493	0.0009	0.0965	0.0004
					N.D: refers to not detected	
**Figure 3b,c**						
	**Average peak area normalized to control (WT, Luciferase)**					
**Compounds**	**Luciferase**	**Luciferase + 10nM FA**	**Luciferase + 10nM 5MTHF**	**Luciferase vs *dhfr-1i***	***dhfr-1i* vs *dhfr-1i* +10nM 5MTHF**	
Methionine	1.0000	0.8567	1.4985	0.5713	0.9839	
S-adenosyl methionine	1.0000	0.7471	1.4166	0.5764	1.1430	
S adenosyl Homocysteine	1.0000	1.4537	0.7469	1.5948	0.6804	
Homocysteine	1.0000	1.4126	0.6632	1.6391	0.4557	
Significance was assessed using one-way anova Dunnett's multiple comparisons test. * p<0.5, ** p<0.01, ***p<0.001						
**Compounds**		**Luciferase vs +10nM FA**	**Luciferase vs +10nM 5MTHF**	**Luciferase vs *dhfr-1i***	***dhfr-1i* vs *dhfr-1i* +10nM 5MTHF**	
Methionine		0.2930	0.0001	0.0005	0.0007	
S-adenosyl methionine		0.0084	0.0001	0.0001	0.0001	
S adenosyl Homocysteine		0.0008	0.0517	0.0001	0.0001	
Homocysteine		0.0001	0.0007	0.0001	0.0001	

**Figure 5a**		
	**Average peak area normalized to control (WT)**	
**Compounds**	**WT**	**daf-2(e1370)**
Methionine	1.0000	0.85179
S-adenosyl methionine	1.0000	0.71733
Homocysteine	1.0000	1.59665
Significance was assessed using two-sided t-test. Bar shows mean ± S.D, * p<0.5, ** p<0.01, ***p<0.001.		
**Compounds**		***daf-2* vs WT**
Methionine		0.1865
S-adenosyl methionine		0.0050
Homocysteine		<0.0001

**Figure 5c**		
Brain	**Average peak area normalized to control (WT)**	
**Compounds**	**WT**	***Irs-1-/-***
FA	1	1.8670
THF	1	2.0140
5MTHF	1	0.2272
ML	1	1.4830
MN	1	0.3860
FO	1	0.6702
Significance was assessed using one-way anova Dunnett's multiple comparisons test. * p<0.5, ** p<0.01, ***p<0.001		
**Compounds**		**Irs-1-/- vs WT**
FA		<0.0001
THF		<0.0001
5MTHF		<0.0001
ML		0.0117
MN		0.0008
FO		0.1681

**Figure 5e**		
Liver	**Average peak area normalized to control (WT)**	
**Compounds**	**WT**	**Irs-1-/-**

FA	1	1.8740
THF	1	2.3910
5MTHF	1	0.2990
ML	1	1.1520
MN	1	0.2509
FO	1	0.4898
Significance was assessed using one-way anova Dunnett's multiple comparisons test. * p<0.5, ** p<0.01, ***p<0.001		
**Compounds**		**Irs-1-/- vs WT**
FA		0.0007
THF		<0.0001
5MTHF		0.0089
ML		0.9778
MN		0.0045
FO		0.1027

**Figure 5d**		
Brain	**Average peak area normalized to control (WT)**	
**Compounds**	**WT**	**Irs-1-/-**
Methionine	1	0.43420
S-adenosylmethionine	1	0.59479
Homocysteine	1	5.33974
Significance was assessed using one-way anova Dunnett's multiple comparisons test. * p<0.5, ** p<0.01, ***p<0.001		
**Compounds**		**Irs-1-/- vs WT**
Methionine		0.0017
S-adenosylmethionine		0.0006
Homocysteine		0.0040

**Figure 5f**		
Liver	**Average peak area normalized to control (WT)**	
**Compounds**	**WT**	**Irs-1-/-**
Methionine	1	0.47328
S-adenosylmethionine	1	0.32255
Homocysteine	1	2.62672

Significance was assessed using one-way anova Dunnett's multiple comparisons test. * p<0.5, ** p<0.01, ***p<0.001

**Compounds**		**Irs-1-/- vs WT**
Methionine		0.0018
S-adenosylmethionine		0.0003
Homocysteine		0.0024

Supplemental Table 7: Lifespan Analyses.

Figure 2a

**Strains**	**Treatment/RNAi**	Mean LS	Mean Diff.	**Max LS**	**Max Diff.**	Median LS	**Median Diff.**	**Log-rank(Mantel-Cox) test**	**Comparison**	**Replicates**
WT	*luciferase*	18.54	0.00%	28	0.00%	17	0.00%			BR1 Shown in Figure 2a
WT	*dhfr-1i*	23.87	28.77%	36	28.57%	24	24.18%	< 0.0001	vs *luciferase*	
WT	*tyms-1i*	22.28	20.18%	36	28.57%	21	23.53%	0.0001	vs *luciferase*	
WT	*daf-2i*	32.58	75.75%	58	107.14%	32	88.24%	< 0.0001	vs *luciferase*	
WT	*luciferase*	16.42	0.00%	30	0.00%	17	0.00%			BR2
WT	*dhfr-1i*	21.57	31.41%	36	20.00%	21	23.53%	0.0002	vs *luciferase*	
WT	*tyms-1i*	21.28	29.65%	34	13.33%	21	23.53%	0.0003	vs *luciferase*	
WT	*daf-2i*	30.18	83.83%	60	100.00%	28	64.71%	< 0.0001	vs *luciferase*	
WT	*luciferase*	16.00	0.00%	28	0.00%	17	0.00%			BR3
WT	*dhfr-1i*	21.36	30.10%	34	21.43%	21	23.53%	0.0002	vs *luciferase*	
WT	*tyms-1i*	20.88	27.18%	36	28.57%	21	23.53%	0.0007	vs *luciferase*	
WT	*daf-2i*	28.74	75.07%	56	100.00%	28	64.71%	< 0.0001	vs *luciferase*	

Figure 2b, Extended data Fig. 3b

**Strains**	**Treatment/RNAi**	Mean LS	Mean Diff.	**Max LS**	**Max Diff.**	Median LS	**Median Diff.**	**Log-rank(Mantel-Cox) test**	**Comparison**	**Replicates**
WT	*luciferase*	21.01	0.00%	29.00	0.00%	23.00	0.00%			BR1 Shown in Figure 2b
WT	*luciferase*+10 nM FA	19.89	-5.34%	29.00	0.00%	21.00	-8.70%	0.0621	vs *luciferase*	
WT	*luciferase*+10 nM 5MTHF	21.01	0.02%	30.00	3.45%	21.00	-8.70%	0.9067	vs *luciferase*	
WT	*dhfr-1i*	25.79	22.75%	39.00	34.48%	25.00	8.70%	<0.0001	vs *luciferase*	
WT	*dhfr-1i*+ 10nM 5MTHF	20.57	-2.10%	29.00	0.00%	21.00	-8.70%	<0.0001	vs *dhfr-1i*	
WT	*dhfr-1i*+ 10nM FA	25.01	19.04%	36	24.14%	22.00	-4.35%	<0.0001	vs *luciferase*	
WT	*luciferase*	20.30	0.00%	29.00	0.00%	19.00	0.00%			BR2

WT	*luciferase*+10 nM FA	19.20	-5.40%	29.00	0.00%	19.00	0.00%	0.238	vs *luciferase*	
WT	*luciferase*+10 nM 5MTHF	20.17	-0.62%	30.00	3.45%	19.00	0.00%	0.5604	vs *luciferase*	
WT	*dhfr-1i*	23.82	17.36%	39.00	34.48%	23.00	21.05%	<0.0001	vs *luciferase*	
WT	*dhfr-1i*+ 10nM 5MTHF	21.19	4.39%	29.00	0.00%	21.00	10.53%	0.0017	vs *dhfr-1i*	
WT	*dhfr-1i*+ 10nM FA	24.50	20.69%	39.00	34.48%	22	15.79%	<0.0001	vs luciferase	
WT	*luciferase*	22.02	0.00%	30.00	0.00%	23.00	0.00%			BR3
WT	*luciferase*+10 nM FA	22.17	0.68%	32.00	0.00%	21.00	0.00%	0.6638	vs *luciferase*	
WT	*luciferase*+10 nM 5MTHF	21.95	-0.30%	32.00	0.00%	21.00	0.00%	0.8677	Vs *luciferase*	
WT	*dhfr-1i*	25.78	22.80%	39.00	21.88%	25.00	8.69%	0.0146	vs *luciferase*	
WT	*dhfr-1i*+ 10nM 5MTHF	20.80	-5.53%	32.00	0.00%	21.00	-8.70%	0.0029	vs *dhfr-1i*	
WT	*dhfr-1i*+ 10nM FA	26.30	19.00%	36	20.00%	25	8.69%	0.0124	vs luciferase	

Figure 3e

**Strains**	**Treatment/RNAi**	Mean LS	Mean Diff.	**Max LS**	**Max Diff.**	Median LS	**Median Diff.**	**Log-rank(Mantel-Cox) test**	**Comparison**	**Replicates**
WT	*luciferase*	24.20		30		28				BR1 Shown in Figure 3e
WT	*luciferase*+ 20mM methionine	23.88	-0.55%	32	6.67%	28	0.00%	0.8479	vs *luciferase*	
WT	*luciferase*+ 40mM methionine	24.20	0.76%	30	0.00%	28	0.00%	0.9103	vs *luciferase*	
WT	*dhfr-1i*	29.70	23.66%	38	26.67%	32	14.29%	<0.0001	vs *luciferase*	
WT	*dhfr-1i*+ 20mM methionine	28.00	16.57%	34	13.33%	30	7.14%	<0.0001	vs *luciferase*	
								0.0078	vs *dhfr-1i*	
WT	*dhfr-1i*+ 20mM methionine	24.10	0.37%	30	0.00%	28	0.00%	0.5484	vs *luciferase*	
								<0.0001	vs *dhfr-1i*	
WT	*luciferase*	22.87		28		24				BR2

WT	*luciferase*+ 20mM methionine	22.46	-1.78%	30	7.14%	24	0.00%	0.3737	vs *luciferase*	
WT	*luciferase*+ 40mM methionine	22.14	-3.19%	28	0.00%	24	0.00%	0.2172	vs *luciferase*	
WT	*dhfr-1i*	28.43	24.32%	36	28.57%	30	25.00%	<0.0001	vs *luciferase*	
WT	*dhfr-1i*+ 20mM methionine	27.005	18.08%	34	21.43%	28	16.67%	<0.0001	vs *luciferase*	
								0.0605	vs *dhfr-1i*	
WT	*dhfr-1i*+ 20mM methionine	22.53	-1.45%	30	7.14%	24	0.00%	0.8635	vs *luciferase*	
								<0.0001	vs *dhfr-1i*	
WT	*luciferase*	22.77		30	0.00%	24				BR3
WT	*luciferase*+ 20mM methionine	22.47	-1.28%	30	0.00%	24	-7.69%	0.3198	vs *luciferase*	
WT	*luciferase*+ 40mM methionine	24.46	7.43%	30	0.00%	26	0.00%	0.4931	vs *luciferase*	
WT	*dhfr-1i*	28.33	24.43%	34	13.33%	30	15.38%	<0.0001	vs *luciferase*	
WT	*dhfr-1i*+ 20mM methionine	26.73	17.41%	32	6.67%	30	15.38%	<0.0001	vs *luciferase*	
								0.0367	vs *dhfr-1i*	
WT	*dhfr-1i*+ 20mM methionine	23.00	1.02%	30	0.00%	26	0.00%	0.8987	vs *luciferase*	
								<0.0001	vs *dhfr-1i*	

**Figure 4a,b**										
**Strains**	**Treatment/RNAi**	Mean LS	Mean Diff.	**Max LS**	**Max Diff.**	Median LS	**Median Diff.**	**Log-rank(Mantel-Cox) test**	**Comparison**	**Replicates**
WT		21.90	0.00%	31.00	0.00%	22.00	0.00%			BR1 Shown in Figure 4a,b
daf-2(e1370)		41.93	91.55%	62.00	100.00%	41.00	86.36%	<0.0001	vs WT	
daf-2(e1370)	+10nM 5MTHF	38.94	77.91%	57.00	83.87%	22.00	77.27%	0.0208	vs *daf-2(e1370)*	
isp-1(qm150)		29.86	36.39%	44.00	41.94%	29.00	31.82%	<0.0001	vs WT	
isp-1(qm150)	+10nM 5MTHF	27.01	23.40%	34.00	9.68%	29.00	31.82%	0.001	vs *isp-1(qm150)*	
WT		23.05	0.00%	31.00	0.00%	24.00	0.00%			BR2
daf-2(e1370)		42.87	86.00%	64.00	106.45%	41.00	70.83%	<0.0001	vs WT	
daf-2(e1370)	+10nM 5MTHF	39.21	70.11%	57.00	83.87%	37.00	54.17%	0.013	vs *daf-2(e1370)*	
isp-1(qm150)		30.15	30.78%	44.00	41.94%	31.00	29.17%	<0.0001	vs WT	
isp-1(qm150)	+10nM 5MTHF	27.19	17.97%	34.00	9.68%	29.00	20.83%	0.0011	vs *isp-1(qm150)*	
WT		20.68	0.00%	31.00	0.00%	24.00	0.00%			BR3
daf-2(e1370)		41.22	99.31%	62.00	100.00%	41.00	70.83%	<0.0001	vs WT	
daf-2(e1370)	*+10nM 5MTHF*	29.21	41.27%	48.00	54.84%	35.00	45.83%	<0.0001	vs *daf-2(e1370)*	
isp-1(qm150)		26.59	28.59%	44.00	41.94%	26.00	8.33%	<0.0001	vs WT	
isp-1(qm150)	*+10nM 5MTHF*	23.51	13.67%	34.00	9.68%	24.00	0.00%	0.0019	vs *isp-1(qm150)*	
**Figure 4e**										
**Strains**	**Treatment/RNAi**	Mean LS	Mean Diff.	**Max LS**	**Max Diff.**	Median LS	**Median Diff.**	**Log-rank(Mantel-Cox) test**	**Comparison**	**Replicates**
WT	*luciferase*	21.67	0.00%	32.00	0.00%	21.00	0.00%			BR1 Shown in Figure 4e
WT	*dhfr-1i*	25.36	17.03%	39.00	21.88%	28.00	33.33%	<0.0001	vs *luciferase*	
daf-16(mgDf50)	*luciferase*	10.48	-51.62%	21.00	-34.38%	9.00	-57.14%	<0.0001	vs *luciferase*	
daf-16(mgDf50)	*luciferase + 10nM 5MTHF*	9.91	-54.27%	23.00	-28.13%	9.00	-57.14%	0.5068	vs *daf-16(mgDf50) luciferase*	
daf-16(mgDf50)	*dhfr-1i*	12.26	-43.40%	25.00	-21.88%	14.00	-33.33%	<0.0001	vs *daf-16(mgDf50) luciferase*	
daf-16(mgDf50)	*dhfr-1i + 10nM 5MTHF*	10.77	-50.27%	23.00	-28.13%	11.00	-47.62%	0.0005	vs *daf-16(mgDf50) dhfr-1i*	

WT	*luciferase*	21.21	0.00%	32.00	0.00%	21.00	0.00%			BR2
WT	*dhfr-1i*	23.51	10.84%	39.00	21.88%	23.00	9.52%	<0.0001	vs *luciferase*	
daf-16(mgDf50)	*luciferase*	11.33	-46.59%	23.00	-28.13%	11.00	-47.62%	<0.0001	vs *luciferase*	
daf-16(mgDf50)	*Luciferase + 10nM 5MTHF*	12.49	-41.12%	23.00	-28.13%	14.00	-33.33%	0.1181	vs *daf-16(mgDf50) luciferase*	
daf-16(mgDf50)	*dhfr-1i*	12.16	-42.69%	25.00	-21.88%	11.00	-47.62%	0.3298	vs *daf-16(mgDf50) luciferase*	
daf-16(mgDf50)	*dhfr-1i + 10nM 5MTHF*	9.77	-53.91%	23.00	-28.13%	9.00	-57.14%	0.9341	vs *daf-16(mgDf50) dhfr-1i*	
WT	*luciferase*	18.94	0.00%	30.00	0.00%	19.00	0.00%			BR3
WT	*dhfr-1i*	22.61	19.62%	35.00	16.67%	23.00	21.05%	0.0029	vs *luciferase*	
daf-16(mgDf50)	*luciferase*	11.35	-39.96%	23.00	-23.33%	11.00	-42.11%	<0.0001	vs *luciferase*	
daf-16(mgDf50)	*luciferase + 10nM 5MTHF*	10.12	-46.43%	23.00	-23.33%	9.00	-52.63%	0.1209	vs *daf-16(mgDf50) luciferase*	
daf-16(mgDf50)	*dhfr-1i*	12.53	-33.69%	25.00	-16.67%	11.00	-42.11%	0.0978	vs *daf-16(mgDf50) luciferase*	
daf-16(mgDf50)	*dhfr-1i + 10nM 5MTHF*	10.72	-43.27%	23.00	-23.33%	9.00	-52.63%	0.0019	vs *daf-16(mgDf50) dhfr-1i*	

Extended Data Fig 3g

Strains	Treatment/RNAi	Mean LS	Mean Diff.	Max LS	Max Diff.	Median LS	Median Diff.	Log-rank(Mantel-Cox) test	Comparison	Replicate
WT	*luciferase*	18.54	0.00%	28	0.00%	17	0.00%			BR1
WT	*mthfr-1i*	20.61	3.28%	30	0.00%	21	0.00%	0.075	vs *luciferase*	
WT	*mel-32i*	17.72	-0.11%	28	0.00%	17	0.00%	0.356	vs *luciferase*	
WT	*tyms-1i*	24.12	28.60%	36	21.43%	24	23.53%	< 0.0001	vs *luciferase*	
WT	*dhfr-1i*	23.89	36.04%	36	28.57%	24	23.53%	< 0.0001	vs *luciferase*	
WT	*F38B6.4i*	17.81	12.89%	28	7.14%	19	0.00%	0.3076	vs *luciferase*	
WT	*daf-2i*	33.68	100.20%	58	114.29%	32	88.24%	< 0.0001	vs *luciferase*	

WT	*dao-3i*	16.05	9.18%	30	0.00%	15	11.76%	0.0718	vs *luciferase*	
WT	*luciferase*	16.98	0.00%	30	0.00%	17	0.00%			BR2
WT	*mthfr-1i*	17.08	0.60%	28	-6.67%	17	0.00%	0.8436	vs *luciferase*	
WT	*mel-32i*	16.52	-2.70%	28	-6.67%	17	0.00%	0.5554	vs *luciferase*	
WT	*tyms-1i*	21.27	25.26%	34	13.33%	21	23.53%	0.0001	vs *luciferase*	
WT	*dhfr-1i*	22.50	32.51%	36	20.00%	21	23.53%	< 0.0001	vs *luciferase*	
WT	*F38B6.4i*	18.67	9.96%	30	0.00%	17	0.00%	0.129	vs *luciferase*	
WT	*daf-2i*	33.11	95.02%	60	100.00%	32	88.24%	< 0.0001	vs *luciferase*	
WT	*dao-3i*	18.06	6.35%	28	-6.67%	19	11.76%	0.583	vs *luciferase*	
WT	*luciferase*	24.54		30		28				BR3
WT	*mthfr-1i*	24.48	0.00%	30	0.00%	28	0.00%	0.8407	vs *luciferase*	
WT	*mel-32i*	24.17	-1.33%	30	0.00%	28	0.00%	0.3837	vs *luciferase*	
WT	*tyms-1i*	29.79	21.62%	36	20.00%	32	14.29%	<0.0001	vs *luciferase*	
WT	*dhfr-1i*	28.56	20.67%	36	20.00%	32	14.29%	<0.0001	vs *luciferase*	
WT	*F38B6.4i*	24.29	-0.84%	30	0.00%	28	0.00%	0.9254	vs *luciferase*	
WT	*daf-2i*	45.97	87.63%	55	83.33%	52	85.71%	<0.0001	vs *luciferase*	
WT	*dao-3i*	25.61	4.56%	32	6.67%	28	0.00%	0.1578	vs *luciferase*	

Extended data Fig. 5d

Strains	Treatment/RNAi	Mean LS	Mean Diff.	Max LS	Max Diff.	Median LS	Median Diff.	Log-rank(Mantel-Cox) test	Comparison	Replicate
WT	*luciferase*	16.25	0%	30	0%	19	0%			BR1
WT	*dhfr-1i*	21.63	33.14%	36	20%	22	15.79%	0.0004	vs *luciferase*	
daf-2(e1370)	*luciferase*	30.35	86.79%	54	80%	32	68.42%	<0.0001	vs *luciferase*	

daf-2(e1370)	*dhfr-1i*	29.56	81.97%	54	80%	30	57.89%	<0.0001	vs *luciferase*	
								0.2610	vs *daf-2(e1370)*	
WT	*luciferase*	18.53	0%	28	0%	17	0%			BR2
WT	*dhfr-1i*	23.91	31.20%	35	25%	22	29.41%	0.0004	vs *luciferase*	
daf-2(e1370)	*luciferase*	31.43	69.59%	58	107.14%	30	76.47%	<0.0001	vs *luciferase*	
daf-2(e1370)	*dhfr-1i*	29.97	61.67%	58	107.14%	30	76.47%	<0.0001	vs *luciferase*	
								0.3458	vs *daf-2(e1370)*	

Supplemental Table 8: List of gene activation upon methionine restriction validated by qRT-PCR according to Tang *et al* (linked to Figure 3d, Figure 5b).

Gene Symbol *H. sapiens*	Gene Title	Delta FC (to the control)	*C. elegans orthologues*	BLAST e value
ERBB2	v-erb-b2 erythroblastic leukemia viral oncogene homolog 2, neuro/glioblastoma derived oncogene homolog (avian)	1.23766075	*let-23*	2.00E-77
DAG1	dystroglycan 1 (dystrophin-associated glycoprotein 1)	1.3569075	*dgn-1*	2.00E-14
PTEN	phosphatase and tensin homolog (mutated in multiple advanced cancers 1)	1.169589	*daf-18*	6.00E-50
DAPK3	death-associated protein kinase 3	2.06537275	*dapk-1*	0
ING2	inhibitor of growth family, member 2	1.16491675	*ing-3*	4.00E-18
TEX14	testis expressed sequence 14 /// testis expressed sequence 14	4.057606	Not found	

Supplemental Table 9: Strains used in this study.

AA numbers/Strains:	Genotype:
	N2 (wild type,* WT*)
**AA3561**	*glp-1 (e2141)ts III*
**CB1370**	*daf-2(e1370)* III
**MQ887**	*isp-1(qm150)* IV
**DA465**	*eat-2(ad465)* II
**AA60**	*daf-16(mgDf50)* I
**AA4776**	N2; *rmIs132[P(unc-54) Q35::YFP*]
**AM141**	*rmIs133[P(unc-54) Q40::YFP*]

Supplemental Table 10: qRT-PCR primers

Gene name	Forward primer sequence	Reverse primer sequence
dhfr-1	TTTCGGAGCCACCATTTGTG	ACCAAGTTTTCTCGCAACGC
mel-32	TTGTTGACTTGCGCCCAATC	TGGGCACGTATTCTTGTTGC
mthfr-1	ATGGGGAAACAGTTCATCGC	TTGATGAACACGCGCTTGAC
dao-3	ATCAATGCTGGACGTTTGGC	TTCGAGCGTCCCAAAACAAC
F38B6.4	AGGTTTCTGCGTTGGCTTTC	AACGTTTGTGGTCCTTTCCG
dapk-1	TGGCAACATTTGTGCACGAG	ACTGTATGCTGAGCAAAGCG
ing-3	TACTGATGACACCGACGTTCTC	ACACCACAAACGCCGAATTC
daf-18	AAATACGGCCGGTAACATGC	TGGTCAACGAAGGCTTTTGC
let-23	CGCTGAAATGGTTGACATGC	TTTGGTTTGCAGCTCGGATG
dgn-1	AAATCAAGAGGACGCAAGCC	AGTGTTGTTGGCTCCACATG
