# Supplementary material for: Regulation of the one carbon folate cycle as a shared metabolic signature of longevity
Source: Nat Commun. 2021 Jun 9;12:3486. doi: 10.1038/s41467-021-23856-9 (PMC8190293; doi:10.1038/s41467-021-23856-9)
Supplement: Supplementary file 3 — Description of Additional Supplementary Information [file 41467_2021_23856_MOESM3_ESM.pdf]

Description of Additional Supplementary Files

File Name: Supplementary Data 1

Description: Metabolomics raw data of longevity mutants, (linked to Figure 1a).

File Name: Supplementary Data 2

Description: Metabolomics raw data of dhfr-1i and metr-1i, (linked to Extended Data Figure 4c).

Metabolomics raw data of dhfr-1i and 5MTHF supplementation, (linked to Extended Data Figure 4a).
